# Depletion of Uhrf1 inhibits chromosomal DNA replication in *Xenopus* egg extracts

**DOI:** 10.1093/nar/gkt549

**Published:** 2013-06-20

**Authors:** Elaine M. Taylor, Nicola M. Bonsu, R. Jordan Price, Howard D. Lindsay

**Affiliations:** ^1^Lancaster Medical School, Faculty of Health and Medicine, Lancaster University, Lancaster, LA1 4YG, UK and ^2^Genome Damage and Stability Centre, University of Sussex, Falmer, Brighton, BN1 9RQ, UK

## Abstract

UHRF1 (ubiquitin-like, containing PHD and RING finger domains 1) has a well-established role in epigenetic regulation through the recognition of various histone marks and interaction with chromatin-modifying proteins. However, its function in regulating cell cycle progression remains poorly understood and has been largely attributed to a role in transcriptional regulation. In this study we have used *Xenopus laevis* egg extracts to analyse Uhrf1 function in DNA replication in the absence of transcriptional influences. We demonstrate that removal of Uhrf1 inhibits chromosomal replication in this system. We further show that this requirement for Uhrf1, or an associated factor, occurs at an early stage of DNA replication and that the consequences of Uhrf1 depletion are not solely due to its role in loading Dnmt1 onto newly replicated DNA. We describe the pattern of Uhrf1 chromatin association before the initiation of DNA replication and show that this reflects functional requirements both before and after origin licensing. Our data demonstrate that the removal of *Xenopus* Uhrf1 influences the chromatin association of key replication proteins and reveal Uhrf1 as an important new factor required for metazoan DNA replication.

## INTRODUCTION

UHRF1 (ubiquitin-like, containing PHD and RING finger domains 1), also called ICBP90 in humans and Np95 in mice, is important for multiple aspects of epigenetic regulation, including maintenance of DNA methylation patterns and recognition of various histone modifications. Several discrete functional domains of UHRF1 are involved in the recognition of specific chromatin modifications. The SRA domain mediates UHRF1 binding to hemimethylated CpG and recruits the maintenance methyltransferase DNMT1 to its hemimethylated DNA substrate (1–5). The tandem Tudor domain directs UHRF1 binding to the heterochromatin mark histone H3K9me3, whereas the PHD domain targets UHRF1 to unmodified histone H3 in euchromatic regions (6–9). UHRF1 also contains a C-terminal RING domain and has been shown to exhibit both autocatalytic E3 ubiquitin (Ub) ligase activity and activity against histone H3 and DNMT1 (10–12). Physical interactions between UHRF1 and various chromatin-modifying cofactors, such as the DNA methyltransferases DNMT1, DNMT3a and DNMT3b; the histone deacetylase HDAC1; the histone methyltransferase G9a and the histone acetyltransferase Tip60, have also been reported, implying a key role for UHRF1 in epigenetic crosstalk (1,2,13–16).

In addition, many studies have correlated UHRF1 expression with cell proliferation. Cell cycle-regulated expression of UHRF1 occurs coincidentally with S phase progression in mouse 3T3 cells (17). Moreover, UHRF1 is upregulated throughout the cell cycle in highly proliferating cells, such as cancer cell lines, primary tumours and pluripotent stem cells, but downregulated during differentiation or quiescence (11,18–21). Depletion of UHRF1 has been shown to reduce the growth rates of several cell types, whereas overexpression of UHRF1 can trigger S phase re-entry in terminally differentiated mouse myotubes and serum-starved human lung fibroblasts (11,13,16,22–25). To date, the effect of UHRF1 on cell cycle progression has largely been ascribed to a role in transcriptional regulation. UHRF1 can function as a transcriptional repressor through its binding to histone H3 when it is unmodified at Arg2 (8). Notably, UHRF1 overexpression in human lung fibroblasts results in downregulation of expression of the tumour suppressor pRB (24). A role for UHRF1 in transcriptional repression of the cell cycle regulator p21 has also been reported (13). UHRF1-dependent repression of factors that serve to restrain the onset of S phase has therefore been proposed to facilitate the G1-S transition. In addition, a direct role for UHRF1 during DNA replication was revealed with the discovery that it recruits DNMT1 to replicating DNA (1,2). This activity is necessary to maintain cytosine methylation patterns, but there is, as yet, little evidence to indicate that this particular function of UHRF1 affects S phase progression. In contrast, siRNA knockdown of mouse UHRF1 has been reported to reduce the replication of pericentric heterochromatin during mid-late S phase (22). It has been suggested that this effect on heterochromatin replication may reflect a role for UHRF1 in inducing a more open chromatin conformation at these highly compacted regions (26).

To further investigate any direct involvement of UHRF1 in DNA replication, beyond the G1–S transition, we have examined UHRF1 function using the *Xenopus laevis* egg extract system, in which DNA replication can be studied in the absence of transcriptional events (27). We describe the regulated chromatin association of *Xenopus* Uhrf1 during S phase and demonstrate that depletion of Uhrf1 inhibits replication of chromosomal DNA in this synchronous cell-free system. We show that Uhrf1 is not needed for DNA synthesis per se, but that Uhrf1, or an as-yet-unidentified Uhrf1-associated factor, is required before replication licensing for efficient chromatin loading of replication proteins, including components of the origin recognition complex (ORC). Furthermore, we show that removal of Uhrf1 additionally affects chromosomal replication at a stage after origin licensing and suggest that this reflects a possible role in maintaining the stable chromatin association of components of the replication complex.

## MATERIALS AND METHODS

### Cloning procedures

tBlastn searches using the human amino acid sequence of UHRF1 identified numerous *Xenopus laevis* expressed sequence tags (ESTs) with significant identity to the human protein. Alignment of the available nucleotide sequences identified a contiguous sequence spanning a predicted open reading frame of 772 amino acids. The complete open reading frame was polymerase chain reaction-amplified from cDNA and cloned into pGEM-Teasy (Promega), whereas 5′ and 3′ fragments of *Xenopus* Uhrf1 were amplified with NdeI/SalI termini and cloned into the expression vector pET16b (Novagen). *Xenopus* Uhrf1 nucleotide and amino acid sequences were deposited in Genbank (Accession number EU177101). An IMAGE cDNA clone (5073177) containing the *Xenopus* homologue of Dnmt1 was identified from the database. Oligonucleotide primers were designed to amplify both the full-length open reading frame and a 3′ fragment of *Xenopus* Dnmt1, which were cloned into pGEM-Teasy and pET16b, respectively.

### Antibodies and reagents

Rabbit polyclonal antibodies were raised (Eurogentec) against recombinant 6xHis-tagged proteins corresponding to the N-terminal 467 aa or the C-terminal 271 aa of *Xenopus* Uhrf1 and to 1201–1490 aa of *Xenopus* Dnmt1. Antibody affinity purification was performed using the respective antigen immobilized on Amino link+ resin (Perbio), according to the manufacturer’s instructions. Antibodies against *Xenopus* Orc1 and Orc2 were a gift from J. Blow (University of Dundee, UK). The *Xenopus* RPA34 antibody was provided by D. Maiorano (IHG, Montpelier, France), and anti-Cdc45 antibody was a gift from V. Costanzo (Clare Hall Laboratories, London, UK). Mouse monoclonal antibody against Mcm7 and rabbit polyclonal anti-histone H3 were purchased from Abcam. Anti-proliferating cell nuclear antigen antibody was from Santa Cruz Biotechnology, anti-histone H2A from Millipore and horseradish peroxidase-conjugated anti-rabbit and anti-mouse secondary antibodies from Dako. Roscovitine was used at a final concentration of 0.5 mM. Caffeine was used at 5 mM. Glutathione-S-transferase-tagged-geminin, expressed in BL21 DE3 cells and purified on glutathione sepharose according to the method of Stokes *et al.* (28), was used at a final concentration of 80 nM. His-Ubiquitin, expressed in BL21 (DE3) pLysS cells and purified on Ni^2+^-NTA agarose (Qiagen), was used at 0.1 mg/ml.

### Preparation of *Xenopus* egg extract

Demembranated sperm nuclei were prepared by lysolecithin treatment, as previously described (29). For preparation of interphase *Xenopus* egg extracts, unfertilized eggs were dejellied, washed and activated with the calcium ionophore A23187, as previously described (30). Activated eggs were washed in extraction buffer (XB: 10 mM 4-(2-hydroxyethyl)-1-piperazineethanesulfonic acid (HEPES-KOH), pH 7.7, 100 mM KCl, 0.1 mM CaCl_2_, 1 mM MgCl_2_, 50 mM sucrose) at 4°C, packed by brief centrifugation at 5000× *g* and then, after removal of excess buffer, crushed by centrifugation at 15 000× *g* for 10 min (4°C). The cytoplasmic layer was supplemented with aprotinin (10 µg/ml), cytochalasin B (50 µg/ml), creatine phosphate (30 mM) and creatine phosphokinase (150 µg/ml), and then centrifuged for 10 min at 60.000 × *g* (4°C) (Beckman Optima, TLA-55) to generate the replication-competent supernatant fraction. Cycloheximide was added to the extract (100 µg/ml), unless otherwise indicated. Replicating extracts were also prepared after the method of Kubota and Takisawa, where indicated (30). In this case, activated eggs were washed, packed and crushed in S buffer (50 mM KCL, 2.5 mM MgCl_2_, 250 mM sucrose, 2 mM β-mercaptoethanol). The cytoplasmic layer was supplemented with cytochalasin B (50 µg/ml) and centrifuged at 208 000 × *g* for 15 min (4°C) (Beckman Optima, TLS-100.3). The supernatant fraction was supplemented with creatine phosphate (30 mM) and creatine phosphokinase (150 µg/ml).

### Nickel–NTA pull-down experiments

Recombinant His-Ubiquitin was added (0.1 mg/ml final concentration) to 25 µl S buffer extract supplemented with nuclei (3000 nuclei µl^−^^1^) before incubation at 21°C. In all, 450 µl of Bind buffer (20 mM 3-(N-morpholino)propanesulfonic acid (MOPS), pH 7.9, 500 mM NaCl, 6 M urea, 16 mM imidazole) was added to the samples, which were then mixed with Ni^2+^-NTA agarose (1.5 h, 4°C) to capture His-tagged proteins. The beads were washed three times with Wash buffer (20 mM MOPS, pH 7.9, 500 mM NaCl, 6 M urea, 30 mM imidazole) before elution of bound proteins in elution buffer (20 mM MOPS, pH 7.9, 500 mM NaCl, 6 M urea, 250 mM imidazole). Sodium dodecyl sulphate (SDS) sample buffer was added, and the eluted proteins were analysed by Western blotting.

### Immunodepletion

*Xenopus* extract was incubated with 40% (v/v) protein A sepharose (GE-Healthcare) cross-linked to affinity-purified Uhrf1or Dnmt1 antibodies for 45 min at 4°C, with occasional resuspension of the beads. Two rounds of depletion were routinely performed, unless otherwise stated. Mock-depleted extracts were treated identically, except protein A sepharose was cross-linked to non-specific rabbit IgGs (Sigma). Antibodies were cross-linked with dimethyl pimelimidate using the method of (31)

### DNA replication assay

DNA replication was measured by α^32 ^P-dCTP incorporation into DNA (32). Extract supplemented with sperm chromatin (3000 nuclei µl^−^^1^) and α^32 ^P-dCTP was incubated at 21°C before addition of replication stop buffer (20 mM Tris, pH8, 30 mM ethylenediaminetetraacetic acid [EDTA], 1% SDS, 600 µg/ml proteinase K). For pulse-label experiments, α^32 ^P-dCTP was added for 10 min. Radionucleotide incorporation was monitored by determining trichloroacetic acid-precipitable counts. The amount of DNA synthesized was calculated using the formula: pg DNA synthesized = (%dCTP incorporated) × (pmoles dCTP in the assay) × 4 × 330, where dCTP concentration in extract is assumed to be 60 pmol/µl.

### Alkaline agarose gel analysis

For alkaline agarose gel analysis, egg extract supplemented with sperm nuclei (3000 nuclei µl^−^^1^) was incubated at 21°C and pulse labelled for 2.5 min with α^32 ^P-dCTP. Replication stop buffer was added and the samples were phenol/chloroform-extracted, ethanol-precipitated and resuspended in alkaline gel-loading buffer (60 mM NaOH, 1 mM EDTA, 3% Ficoll, 0.0125% bromophenol blue). Agarose gels (0.8%) were prepared in 50 mM NaCl, 1 mM EDTA and then equilibrated in alkaline running buffer (30 mM NaOH, 2 mM EDTA) for 3 h. Gels were run for 16 h at 2 V cm^−^^1^ and then fixed in 7% trichloroacetic acid and 1.4% sodium pyrophosphate for 20 min before being dried and exposed to a phosphorimager screen.

### Chromatin isolation and chromatin transfer

For chromatin isolation, 25 -µl extract containing sperm chromatin (3000 nuclei µl^−^^1^) was diluted with 200 µl of XB containing 0.25% Triton X-100 and centrifuged through 800 µl of 750 mM sucrose (in XB) at 5000 × *g* (5 min, 4°C). After washing the cytoplasmic/sucrose interface twice with XB/Triton X-100, the supernatant was removed and the chromatin pellet was washed with XB/Triton X-100. After centrifuging at 10 000 × *g* (5 min, 4°C), the wash buffer was removed and the chromatin pellet resuspended in SDS-polyacrylamide gel electrophoresis buffer. For chromatin transfer experiments, 40 -µl extract containing sperm chromatin (4000 nuclei µl^−^^1^) was diluted in XB containing 15 mM MgCl_2_ and 0.08% Triton X-100 and centrifuged through a 750-mM sucrose layer at 5000 × *g* (5 min, 4°C). Chromatin pellets were washed once with XB/15 mM MgCl_2_ and resuspended in 25 µl of egg extract.

## RESULTS

### Cell cycle-dependent chromatin association and ubiquitylation of Uhrf1

**Figure 1.**
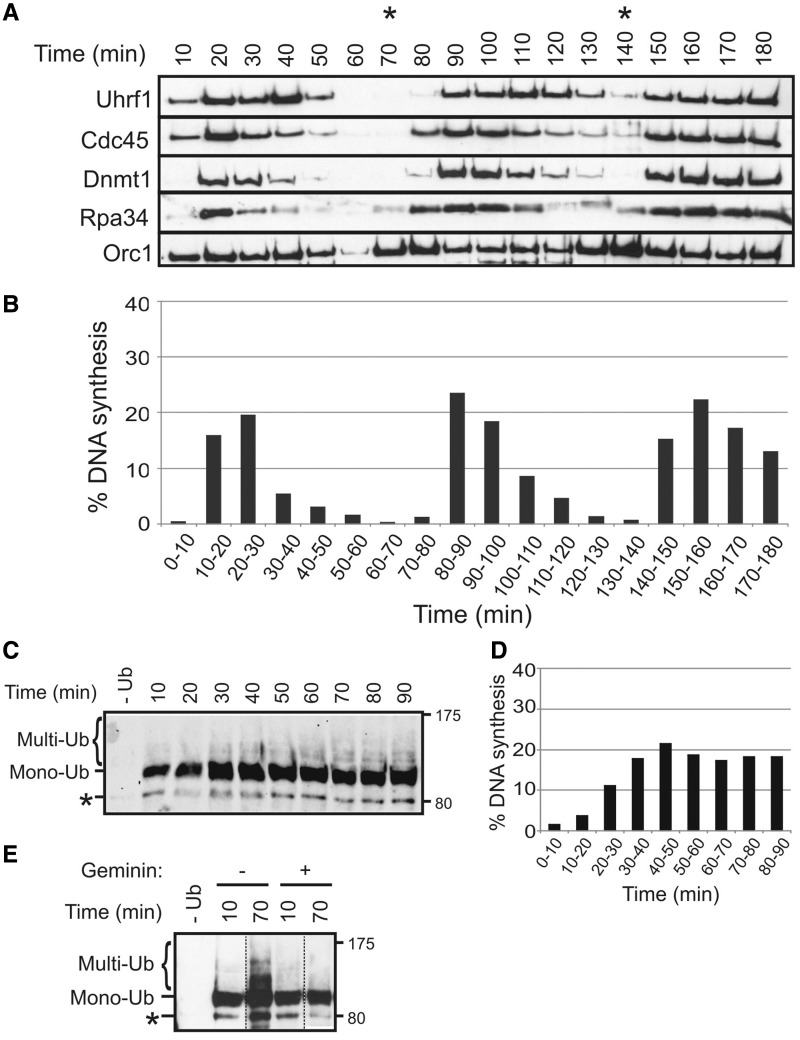
Uhrf1 associates with chromatin and undergoes ubiquitylation during DNA replication. (**A**) XB extract, without cycloheximide, was supplemented with sperm chromatin and incubated at 21°C. Chromatin was isolated at 10-min intervals and associated proteins were immunoblotted with the indicated antibodies. Orc1 serves as a loading control. The timing of mitosis is indicated by asterisk. (**B**) Pulse-label DNA replication assay performed alongside chromatin time-course in (A). (**C**) Egg extract (S buffer) was supplemented with recombinant His-ubiquitin (0.1 µg/µl) and sperm nuclei and incubated at 21°C. At the indicated times, samples were purified on nickel agarose and eluted proteins were immunoblotted with Uhrf1 antibody. (**D**) Pulse-label DNA replication assay associated with ubiquitylation time-course in (C). (**E**) His-Ub (0.1 µg/µl) and geminin (80 nM) were added to egg extract (S buffer) containing nuclei, as indicated. Samples were purified on nickel agarose after 10 min and 70 min and immunoblotted for Uhrf1. Dotted lines indicate removal of intervening lanes on the same gel for the sake of clarity. Probable monoubiquitylated Uhrf1 degradation product is indicated by asterisk.

To study UHRF1 function in DNA replication using *Xenopus* egg cell-free extracts, we first isolated the *Xenopus* orthologue of UHRF1. *Xenopus* Uhrf1 cDNA encodes a 772 aa protein that is 66% identical to the human protein and shares an equivalent domain structure. Polyclonal antibodies raised to either the N- or C-terminus of Uhrf1 each recognize a single 80-kDa band in *Xenopus* egg extract, corresponding to the Uhrf1 protein (Supplementary Figure S1). Using these antibodies, we examined the chromatin association of Uhrf1, alongside that of known replication factors, throughout the cell cycle (Figure 1A and B). Orc1 was rapidly loaded onto the DNA and remained chromatin-associated through three rounds of DNA replication (33). In contrast, Uhrf1, along with the replication proteins Cdc45, hypophosphorylated RPA34 and Dnmt1, all exhibited a cyclical pattern of chromatin binding during DNA replication and were largely displaced at the end of each S phase (34,35). Further examination of the chromatin samples revealed the appearance of Uhrf1-degradation products during DNA replication, peaking at mid-late S phase (Supplementary Figure S2). These data suggest that proteolytic degradation may contribute to the removal of chromatin-associated Uhrf1 as S phase proceeds, although total Uhrf1 levels in egg extract do not vary noticeably through the cell cycle (Supplementary Figure S2).

Mammalian UHRF1 homologues possess E3 Ub ligase activity and undergo autoubiquitylation (10,36). Moreover, ubiquitylation of human UHRF1 leads to its proteasomal degradation during mitosis, and this protein turnover is important in regulating cell proliferation (37). We therefore asked whether Uhrf1 became similarly ubiquitylated in *Xenopus* egg extracts. After the addition of recombinant His-Ub to egg extract, we were able to purify both monoubiquitylated and, to a lesser extent, multiple ubiquitylated forms of Uhrf1 on nickel agarose. Uhrf1 monoubiquitylation occurred relatively quickly and was apparent throughout the cell cycle, whereas multiple ubiquitylated Uhrf1 was only detected during the S phase, coincident with the appearance of Uhrf1-degradation products (Figure 1C–E). Moreover, inhibition of DNA replication by addition of geminin had no effect on Uhrf1 monoubiquitylation but completely abolished further ubiquitylation, demonstrating the DNA replication-dependence of this event. As we do not readily detect ubiquitylated Uhrf1 in egg extract, without first enriching for His-ubiquitylated proteins, it is clear that only a fraction of the *Xenopus* Uhrf1 is modified under these conditions. However, modification of only a small proportion of a target protein is a common feature of many ubiquitin and ubiquitin-like modification pathways (38). Together these findings confirm that Uhrf1 is a target of ubiquitin modification in *Xenopus* egg extracts, as it is in mammalian cells, and suggest that Ub-dependent proteolysis may be a conserved mechanism for removal of this protein after it has completed its function during the S-phase.

### Uhrf1 depletion inhibits chromosomal replication

**Figure 2.**
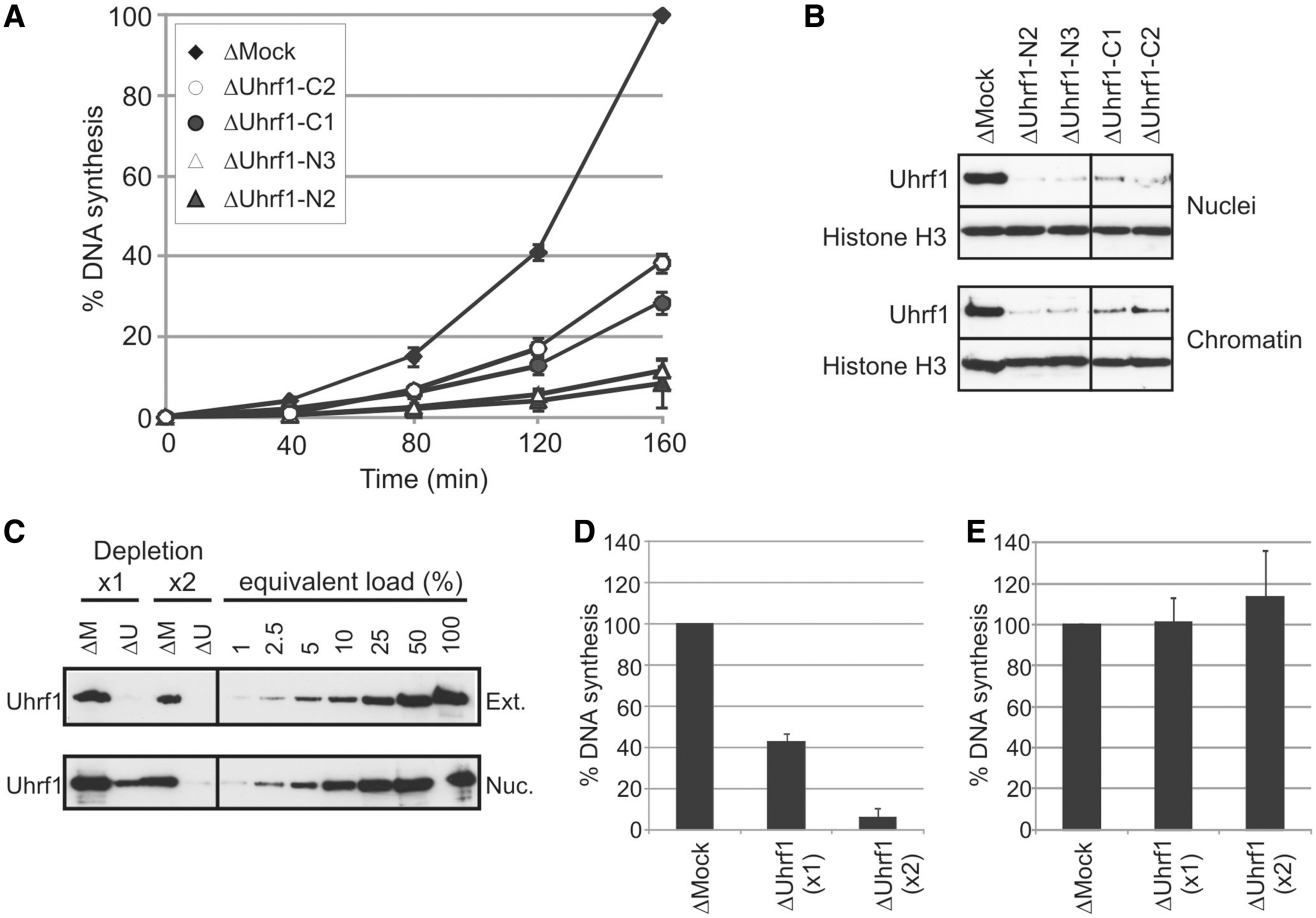
Uhrf1 is required for efficient DNA replication in *Xenopus* egg extract. (**A**) XB extract was subjected to two rounds of immunodepletion using sepharose beads cross-linked to either non-specific IgG (Mock) or to antibodies raised to the N-terminus (N2 and N3) or C-terminus (C1 and C2) of Uhrf1. DNA replication in each extract was measured by continuous incorporation of α-^32^PdCTP into sperm chromatin. (**B**) Western blot of Uhrf1 in nuclear and chromatin fractions after depletion with the indicated antibody beads. Histone H3 serves as a loading control. (**C**) Western blot comparing Uhrf1 levels in cytoplasmic extract and nuclei after one (×1) or two (×2) rounds of depletion with control beads (ΔM) or anti-Uhrf1-N2 antibody beads (ΔU), alongside dilution ranges of mock-depleted extract/nuclear fractions (where 100% is an equivalent load to ΔM ×1 depletion) (**D**) Replication of sperm DNA in XB extract after two rounds of depletion with control beads (ΔMock) or one (×1) or two (×2) rounds of depletion with anti-Uhrf1-N2 beads. (**E**) DNA synthesis on M13 ssDNA template in ΔMock and ΔUhrf1 extracts (one or two rounds of depletion, as indicated). In each case, the data represent three independent experiments and error bars indicate standard deviation (SD).

To determine whether the cell cycle-regulated chromatin association of Uhrf1 reflects a functional role in DNA replication, we examined the replication proficiency of egg extract after immunodepletion of Uhrf1. Strikingly, DNA replication was inhibited when Uhrf1 was removed from egg extract using various different anti-Uhrf1 antibodies (Figure 2A). DNA replication was effectively abolished when Uhrf1 was immunodepleted using polyclonal antibodies to the N-terminus of the protein. Immunodepletion by antibodies to a non-overlapping C-terminal region of Uhrf1 also impaired replication, albeit to a lesser degree (30–40% replication relative to mock-depleted extract). This decreased inhibition of replication correlates with reduced effectiveness of depletion by the C-terminal antibodies, as fractionally more Uhrf1 accumulates in nuclei and on chromatin under these depletion conditions (Figure 2B). To confirm that this very low residual level of Uhrf1 is indeed sufficient to support DNA replication to the extent seen in these α-Uhrf1-C1- and C2-depleted extracts, we further examined the replicative capacity of egg extract after different degrees of immunodepletion. A single round of depletion using the N-terminal antibody α-Uhrf1-N2 was sufficient to reduce Uhrf1 in egg extract to <1% of normal levels (Figure 2C, lane2, top). However, even at these very low amounts, a significant proportion of the remaining protein was accumulated in nuclei, to approximately 5–10% of normal levels (Figure 2C, lane 2, bottom), and supported DNA replication to approximately 40% of mock-depleted levels (Figure 2D), comparable with α-Uhrf1-C1/C2-depleted extracts. In contrast, reducing nuclear Uhrf1 levels by 99% through a further round of depletion prevented almost all replication (Figure 2C, lane 4 and Figure 2D). Importantly though, Uhrf1-depleted extract does still retain the capacity for origin-independent replication of single-stranded M13 DNA, indicating that Uhrf1 is not required for DNA synthesis per se (Figure 4D) (39).

### An early stage in chromosomal DNA replication is affected by Uhrf1 depletion

**Figure 3.**
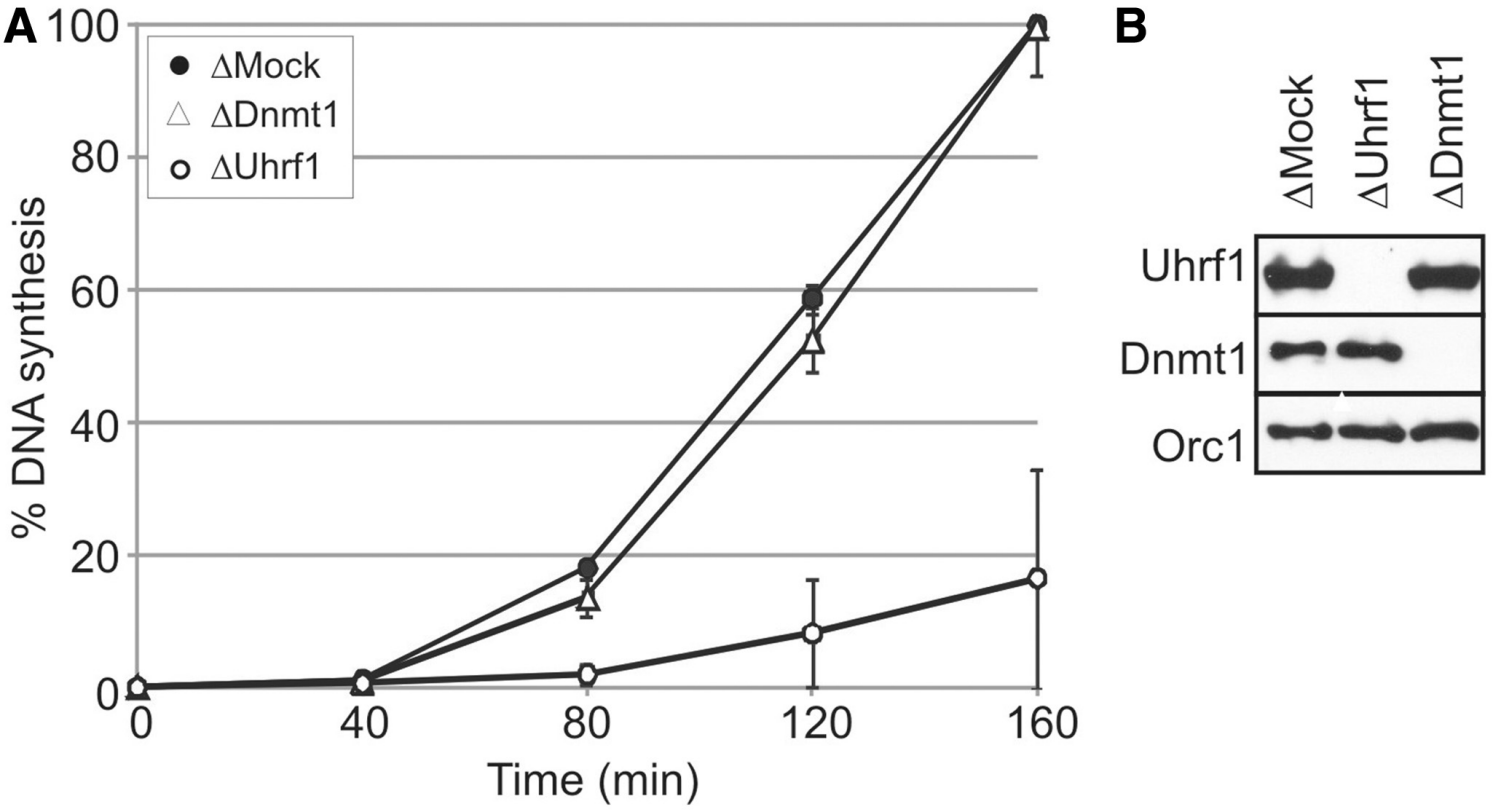
The Uhrf1-dependent replication defect does not reflect a requirement for Dnmt1 loading. (**A**) Mock-, Dnmt1- and Uhrf1-depleted extracts supplemented with sperm chromatin were incubated at 21°C, and DNA replication was measured by continuous incorporation of α-^32^PdCTP. Data from three independent experiments are presented. (**B**) Western blot indicating levels of Uhrf1 and Dnmt1 in depleted and mock-depleted extracts relative to Orc1 loading control.

Because mammalian UHRF1 targets DNMT1 to newly replicated DNA, we reasoned that reduced DNA replication in Uhrf1-depleted *Xenopus* egg extracts could be a consequence of perturbing Dnmt1 loading (1,2). In mammalian cells, UHRF1 and DNMT1 have been shown to interact, but it has been noted that this interaction is stabilized on chromatin and can barely be detected in the absence of DNA (40). It is not surprising then, that in *Xenopus* egg extract in the absence of exogenously added DNA, Dnmt1 is not associated with Uhrf1. We were therefore able to immunodeplete either Uhrf1 or Dnmt1 individually and assess the effects of their removal on DNA replication (Figure 3A and B). It is clear from these data that Dnmt1-depleted extract was fully proficient for DNA replication, whereas Uhrf1-depleted extract was significantly impaired. This indicates that the requirement for *Xenopus* Uhrf1 in DNA replication is not due to its role in loading Dnmt1 onto DNA, as the removal of Dnmt1 has no discernible effect on DNA replication levels in this biochemical model.

**Figure 4.**
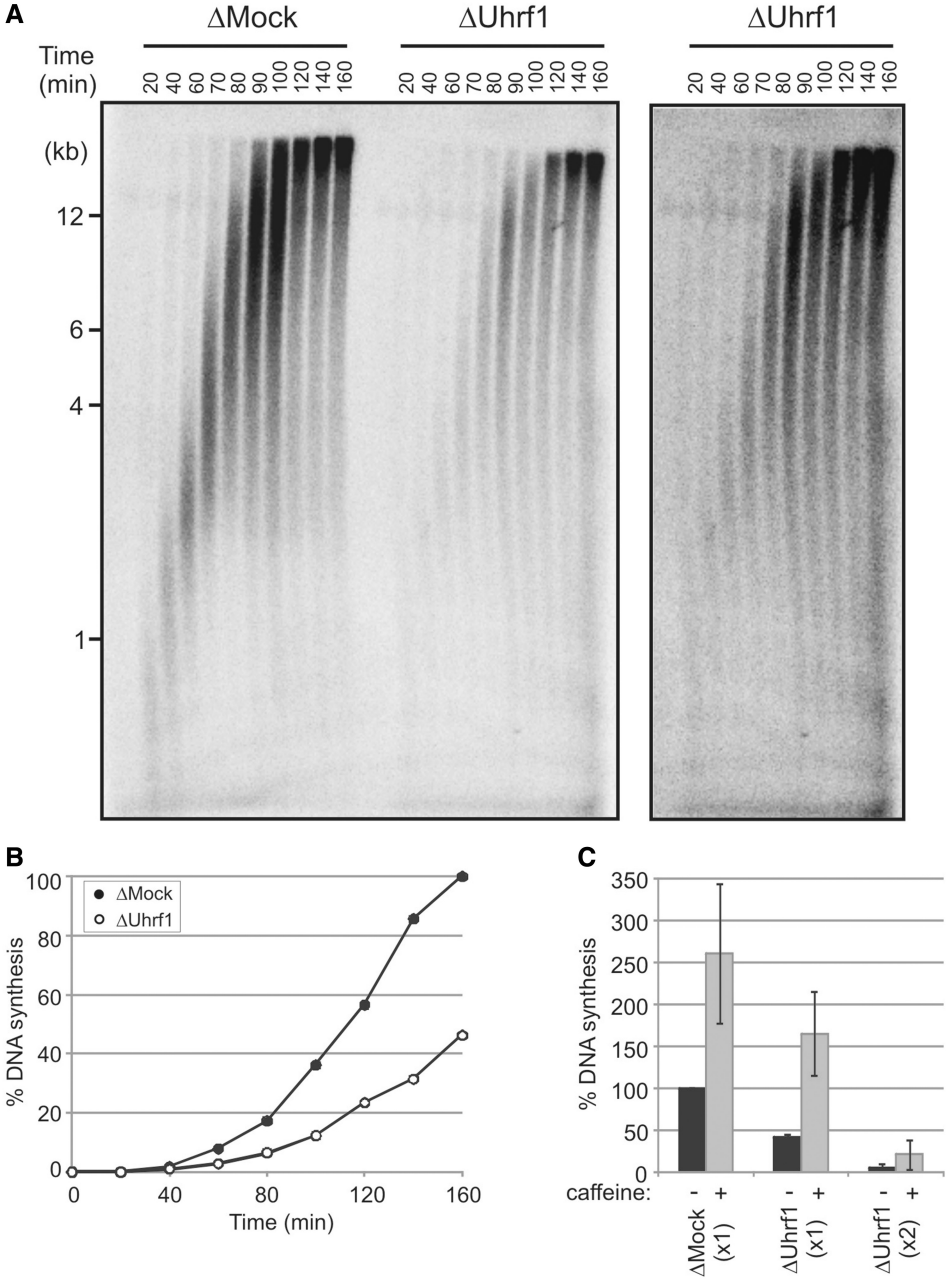
Uhrf1 is required at an early stage of chromosomal DNA replication. (**A**) Mock- and Uhrf1-depleted extracts containing sperm chromatin were pulse labelled with α-^32^PdCTP and radiolabelled DNA replication intermediates were separated on alkaline agarose gels before detection by autoradiography. Panel on right shows a higher exposure of the ΔUhrf1 samples indicating DNA fragment sizes comparable with the ΔMock samples at the same time points. (**B**) DNA replication assay on the ΔMock and ΔUhrf1 extract from (A). (**C**) Replication of sperm chromatin in ΔMock and ΔUhrf1 extracts in the absence or presence of 5 mM caffeine (one or two rounds of depletion, as indicated). Data represent three independent experiments, error bars indicate SD.

To gain further insight as to the nature of the Uhrf1-dependent replication defect, we used alkaline gel electrophoresis to monitor the progress of DNA replication in both mock- and Uhrf1-depleted extracts. Alkaline gel electrophoresis allows the visualization of DNA replication intermediates after pulse-label incorporation of α^32 ^P-dCTP into the nascent DNA strand. Given that near-complete removal of Uhrf1 ablates DNA replication almost entirely, replication intermediates are not formed to any appreciable extent under these conditions. Therefore, we monitored the formation of replication products in partially Uhrf1-depleted extract, which supported replication to <50% of that in mock-depleted extract (Figure 4A and B). From this analysis, it was clear that the total amount of DNA synthesis was reduced after partial depletion of Uhrf1, as previously noted, but interestingly, replication intermediates of increasing length were formed with comparable kinetics in Uhrf1- and mock-depleted extracts. In both ΔMock and ΔUhrf1 samples, DNA fragments of approximately 2–4 kb are apparent by 60 min, 4–12 kb by 80 min and full-length products by 120 min. As decreased fork speeds would lead to a reduction in nascent strand length, these data demonstrate that Uhrf1 depletion, while reducing total DNA replication levels, does not significantly impair the elongation stage of replication. Instead, the data suggest that removal of Uhrf1 leads to a reduced level of origin firing (41). In further support of this interpretation, it is apparent that the replication defect resulting from a single round of Uhrf1 depletion could be rescued by the addition of caffeine, which overrides the intra-S phase checkpoint and activates dormant origins (Figure 4C) (41). However, when Uhrf1 was almost entirely removed, through two rounds of depletion, caffeine could not overcome the block to replication, suggesting that competent origins may not be established under these conditions.

### Uhrf1 depletion impairs ORC loading

**Figure 5.**
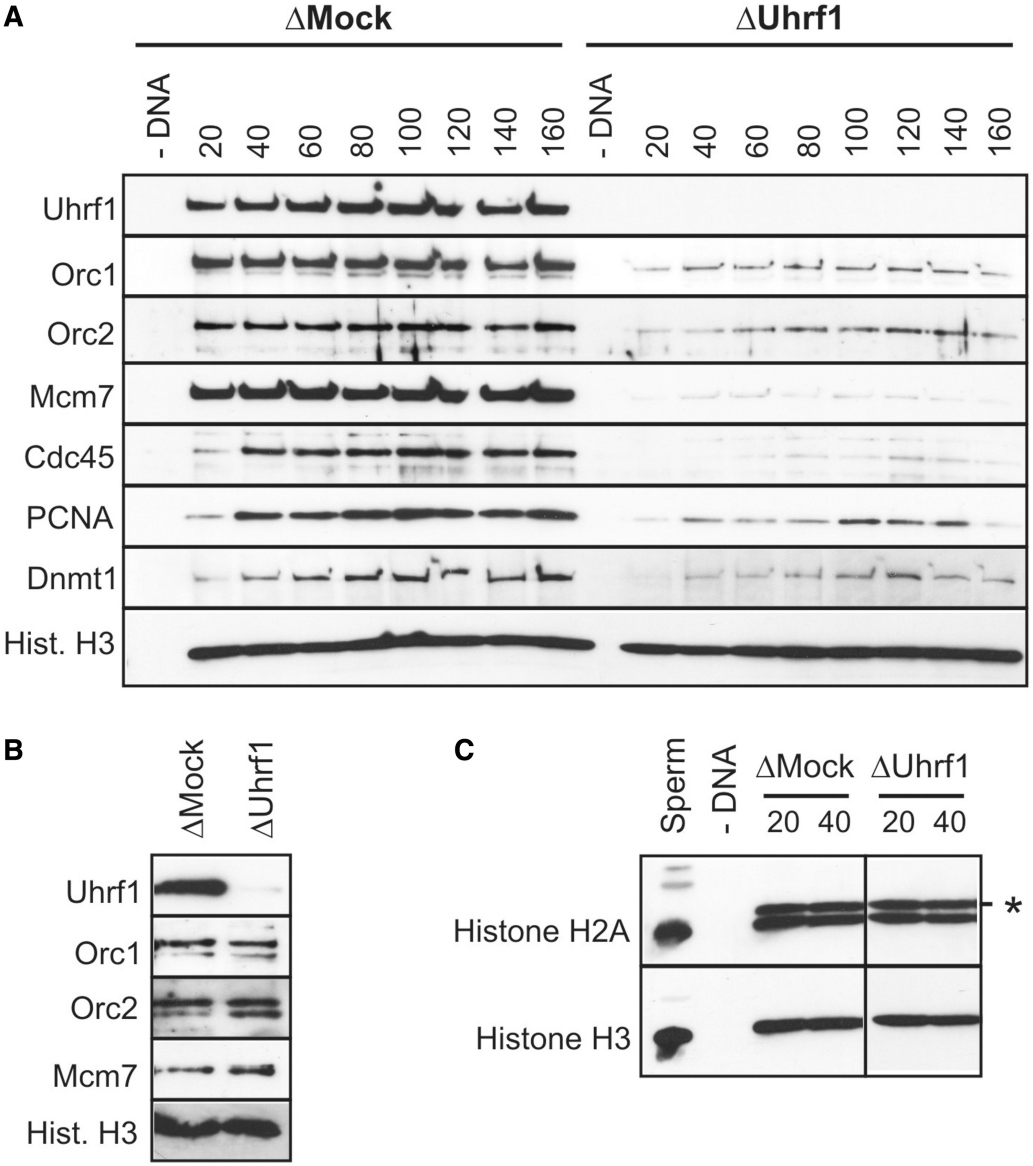
Uhrf1 is required for ORC chromatin loading. (**A**) Sperm chromatin was incubated in ΔMock or ΔUhrf1 extracts for the indicated times and chromatin association of replication proteins was analysed by immunoblotting. (**B**) Western blot to show levels of the indicated replication proteins in nuclei isolated from ΔMock or ΔUhrf1 extracts. (**C**) Sperm chromatin and chromatin isolated from ΔMock or ΔUhrf1 extracts was immunoblotted with antibodies against histone H2A and histone H3. Phosphorylated histone H2A is indicated by asterisk. Histone H3 serves as a loading control.

To investigate how Uhrf1 may influence the production of replication-competent DNA structures, we examined the chromatin binding of a number of DNA replication proteins after Uhrf1 depletion. Remarkably, we noted a marked reduction in chromatin binding for all the replication proteins analysed, including components of the ORC complex, Orc1 and Orc2 (Figure 5A). The amount of nuclear Orc1/Orc2 is unaltered by Uhrf1 depletion, confirming that they are not co-depleted with Uhrf1 and that their nuclear localization is not affected (Figure 5B). Taken together, these data demonstrate that Uhrf1 is important for efficient recruitment of the ORC complex to sperm DNA. Sperm chromatin undergoes extensive nucleoplasmin-dependent remodelling on incubation in *Xenopus* egg extract, resulting in chromatin decondensation, displacement of sperm-specific protamines, the incorporation of core histones H2A/H2B and phosphorylation of histones H2A and H4 (42,43). This remodelling process is known to be important for ORC recruitment (44). However, the reduced ORC loading in Uhrf1-depleted extract does not seem to reflect a general failure of sperm chromatin remodelling under these conditions, as histone H2A is recruited and modified to a comparable degree in both Uhrf1- and mock-depleted extract, and we have not observed any effects on gross nuclear morphology/sperm chromatin decondensation under these conditions (Figure 5C and Supplementary Figure S3).

### Removal of Uhrf1 influences chromosomal replication both before and after replication licensing

**Figure 6.**
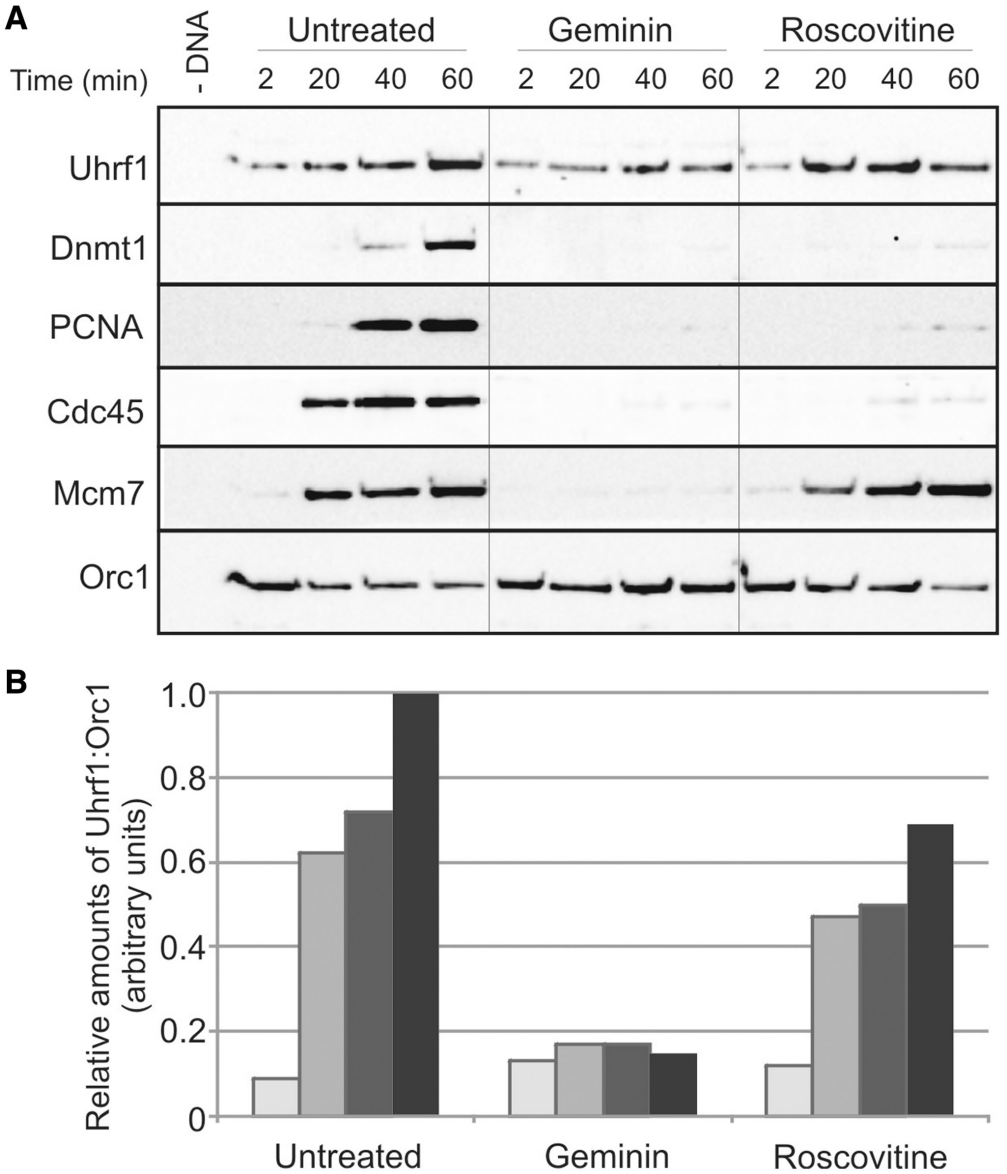
Uhrf1 exhibits two phases of chromatin binding. (**A**) XB extract containing sperm nuclei was incubated at 21°C. Replication inhibitors, geminin (80 nM) and roscovitine, were included as shown. Chromatin was isolated at the times indicated and associated proteins detected by Western blotting. Orc1 serves as a loading control. (**B**) A quantification of the Uhrf1 signal relative to Orc1 loading control is presented.

As our findings imply an early role for Uhrf1 in DNA replication, upstream of ORC loading, we wanted to determine more precisely whether the timing of Uhrf1 chromatin association was consistent with such a function. We therefore examined Uhrf1 chromatin binding in extract containing inhibitors that act at different stages of replication—geminin inhibits origin licensing, whereas the cyclin-dependent kinase (CDK) inhibitor roscovitine allows replication licensing to occur but prevents initiation of replication (Figure 6). From this analysis, it was clear that a proportion of Uhrf1 is loaded onto chromatin very rapidly in a manner that is independent of replication licensing (+ geminin), whereas a further population of Uhrf1 accumulates on chromatin after origin licensing has occurred (untreated extract, + roscovitine). This accumulation is not dependent on the initiation of replication because, like minichromosome maintenance protein (MCM) loading, it occurs in roscovitine-treated extract. This pattern of pre- and post-licensing chromatin association is entirely consistent with a proposed early function for Uhrf1 in ORC loading, and suggests that Uhrf1 may additionally be required at a subsequent stage of DNA replication.

**Figure 7.**
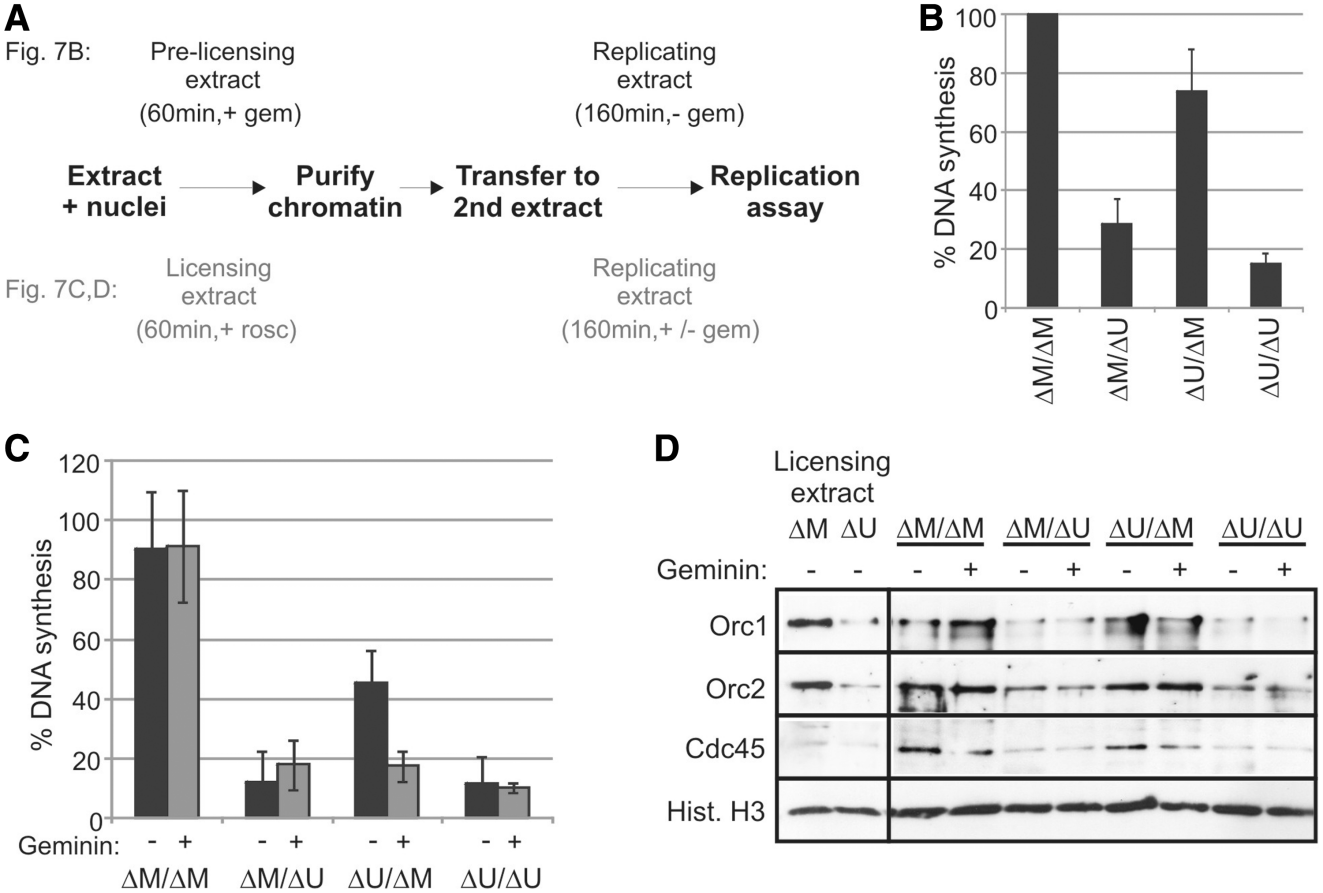
Uhrf1 is required before and after origin licensing. (**A**) A schematic representation of the experiments presented in (**B**) (top) and (**C**) (bottom). (B) Sperm nuclei were incubated for 60 min in ΔMock or ΔUhrf1 extracts containing geminin, then isolated and transferred to fresh ΔMock or ΔUhrf1 extract. DNA replication in the second extract was measured by continuous incorporation of α-^32^PdCTP. Data are from three independent experiments. (C) Sperm nuclei were incubated for 60 min in ΔMock or ΔUhrf1 extracts containing roscovitine before chromatin isolation and transfer to ΔMock or ΔUhrf1 extracts in either the absence or presence of geminin. DNA replication in the second extract was assayed after 160 min. Data represent three independent experiments, error bars indicate SD. (**D**) Chromatin from (C) was isolated after 120-min incubation in second extract and immunoblotted with the indicated antibodies. Left panel shows chromatin isolated after incubation in first extract (ΔMock or ΔUhrf1) alone.

Because depletion of Uhrf1 from *Xenopus* egg extract blocks DNA replication before ORC loading, this serves to mask any additional downstream requirement for Uhrf1. Therefore, to investigate a potential role for Uhrf1 beyond initial ORC loading, we conducted chromatin transfer experiments in which chromatin was isolated from one extract, blocked at an early stage of replication, and transferred to a second extract to complete replication. First, we incubated sperm DNA in depleted extracts containing geminin, to inhibit origin licensing, before transferring the chromatin to a second Uhrf1- or mock-depleted extract (Figure 7A and B). As expected, chromatin transferred between mock-depleted starting and replicating extracts (ΔM/ΔM) was effectively replicated, whereas chromatin transferred between Uhrf1-depleted extracts (ΔU/ΔU) was not. Chromatin transfer from ΔUhrf1 pre-licensing extract to ΔMock extract (ΔU/ΔM) resulted in a substantial level of replication, as, in this instance, the second extract could support both pre- and post-licensing association of Uhrf1. In contrast, chromatin transfer from ΔMock pre-licensing extract to ΔUhrf1 extract (ΔM/ΔU) did not lead to DNA replication. In this case, the starting extract would support the initial pre-licensing association of Uhrf1 but any further accumulation of Uhrf1 after origin licensing would be prevented. The inhibition of DNA replication under these conditions indicates a post-licensing requirement for Uhrf1 in this process.

In a second set of experiments, we used roscovitine to block initiation in the starting extracts before chromatin transfer to a second extract (untreated or treated with geminin to prevent new origin licensing) (Figure 7A, C and D). Once again, transfer between mock-depleted extracts (ΔM/ΔM) resulted in DNA replication, and the inclusion of geminin had little effect, as origins were already efficiently licensed in the starting extract. Chromatin transfer from ΔUhrf1 to ΔMock extract (ΔU/ΔM) also facilitated DNA replication (50%), as the second extract could provide pre- and post-licensing Uhrf1 functions. In this instance, however, geminin did inhibit replication, by preventing licensing of newly established ORC bound-origins in the second extract. Intriguingly, chromatin that had set up licensed origins in ΔMock extract before transfer to ΔUhrf1 extract (ΔM/ΔU) did not replicate to any significant degree, indicating a Uhrf1-dependent function after the roscovitine restriction point, at or post-initiation.

Because our alkaline gel analysis of replication intermediates did not indicate any major requirement for Uhrf1 in the elongation step of DNA replication, we considered the possibility that Uhrf1 involvement at or beyond initiation may relate to maintenance of the pre-replication/pre-initiation complex (pre-RC/IC) at licensed origins. We therefore examined the chromatin association of pre-RC/IC components after chromatin transfer (Figure 7D). After initial incubation in roscovitine-treated starting extracts, we observed significant ORC binding to ΔMock chromatin, little ORC binding to ΔUhrf1 chromatin and little Cdc45 chromatin association in either starting extract, as anticipated (Figure 7D, left panel). Also as expected, Orc1, Orc2 and Cdc45 were readily detected on chromatin after transfer between ΔMock extracts (ΔM/ΔM), but reduced on chromatin from ΔUhrf1 starting/replicating extracts (ΔU/ΔU). All three proteins were bound to chromatin transferred from ΔUhrf1 to ΔMock extract (ΔU/ΔM), but Cdc45 loading (not Orc1/2) was somewhat reduced by geminin-dependent inhibition of licensing in the second extract, as expected. Notably, chromatin that was isolated from ΔMock/ΔUhrf1 chromatin transfer (ΔM/ΔU) exhibited a significant reduction in Orc1, Orc2 and Cdc45 binding relative to ΔM/ΔM chromatin. This suggests that the pre-RC established in roscovitine-blocked ΔMock extract (Figure 7D, left panel) was destabilized on transfer to Uhrf1-depleted extract. These findings imply an ongoing requirement for Uhrf1 to maintain the pre-RC at replication origins until after initiation of DNA replication.

## DISCUSSION

The correlation between UHRF1 expression and cell proliferation is well established and has largely been attributed to a role for UHRF1 in transcriptional regulation of the G1–S transition. However, several studies raise the possibility that UHRF1 has a more direct involvement in DNA replication. For example, mouse UHRF1 co-localizes with the replication factor proliferating cell nuclear antigen during early-mid S phase, although the divergence of their localization patterns, later in S phase, has been taken to indicate that UHRF1 is not an integral part of the replication machinery itself (19). Co-localization of UHRF1 with the maintenance methyltransferase DNMT1 has also been described in mid-late S phase, reflecting its role in the recruitment of DNMT1 to newly replicated hemi-methylated DNA (1,2). This activity is required for the faithful inheritance of DNA methylation patterns. In addition, UHRF1, targeted to heterochromatin through its binding to histone H3K9me3, is required for the timely replication of pericentric heterochromatin, perhaps reflecting a role for UHRF1 in opening up the chromatin structure of these highly compacted regions (22,26).

To further elucidate its involvement in DNA replication, we have used a well-characterized replication model, free from transcriptional regulation, to study Uhrf1 function. We found that *Xenopus* Uhrf1 accumulates on chromatin during DNA replication but is largely displaced from chromatin at the end of S phase. This pattern of association is seen in many proteins that function during DNA replication and are then removed from DNA to re-establish replication-proficient conditions in the subsequent cell cycle. Replication-dependent ubiquitylation and proteolytic degradation of chromatin-bound Uhrf1 as S phase progresses suggests that Ub-dependent proteolysis may be an important mechanism for the removal of chromatin-associated Uhrf1 once it has completed its function. These data mirror the findings that in human cells, UHRF1 is ubiquitylated and degraded during mitosis (37). Moreover, there is some evidence that this protein turnover is important for cell proliferation, as human cells expressing a stable UHRF1 mutant protein exhibit a reduced growth rate.

From our immunodepletion studies, it is evident that removal of Uhrf1 prevents efficient chromosomal replication in *Xenopus* egg extract. The degree of replication inhibition correlates with the extent of Uhrf1 depletion and is reproducible using any of several different antibodies raised to different Uhrf1 epitopes, strongly supporting the Uhrf1-dependent nature of this effect. However, addition of recombinant Uhrf1 does not rescue this replication activity, and so we are not able to demonstrate unequivocally whether Uhrf1 is the only factor required. It is equally possible that co-immunodepletion of an as-yet-unidentified Uhrf1-associated protein gives rise to this effect on chromosomal replication. This is especially the case, given that Uhrf1 exists in a high molecular weight complex in *Xenopus* egg extract, although to date, we have been unable to identify any co-immunoprecipitating partners with known functions in DNA replication or chromatin remodelling (data not shown). Although interactions between Uhrf1 and a variety of proteins involved in chromatin remodelling have been reported in mammalian cells, it is likely that many of these interactions occur in the context of chromatin. It is therefore not surprising to find that these interactions are not detectably recapitulated in *Xenopus* egg extract in the absence of exogenously added DNA.

The replicative requirement for Uhrf1, and/or a co-depleting partner protein, is not due to its role in loading Dnmt1 onto newly synthesized DNA and neither does it reflect a role in the replication of non-chromatinized templates or during the elongation step of DNA synthesis. Instead, our data indicate a requirement during an early stage of chromosomal replication. In fact, the two-stage chromatin binding of Uhrf1, both before and after replication licensing, points towards two phases of Uhrf1 involvement in this process. We have demonstrated that Uhrf1 depletion significantly impairs the chromatin binding of a number of replication proteins, including components of the ORC complex that normally associate with DNA before replication licensing. In addition, chromatin transfer experiments indicate further involvement after origin licensing, and may be important for the maintenance of ORC chromatin association until DNA replication has been initiated. Nucleoplasmin-dependent remodelling of sperm chromatin is known to be important for ORC recruitment in *Xenopus* egg extracts, but our data do not indicate a gross failure of sperm chromatin remodelling after Uhrf1-depletion (44). Moreover, our chromatin transfer data confirm a requirement for Uhrf1 in chromosomal replication even after sperm chromatin has been unpackaged.

Although chromosomal replication is abolished after immunodepletion of Uhrf1, it is clear that, after partial depletion, some DNA replication can still occur at surprisingly low levels of Uhrf1. This fact may help to account for some of the experimental variation observed using siRNA knockdown approaches in mammalian cells. It has previously been noted that the consequences of UHRF1 depletion for cell proliferation are rather variable depending on the cell model used (45). This may reflect the degree of UHRF1 depletion achieved in each case as well as possible cell type-specific differences. Deletion of UHRF1 causes early embryonic lethality, in both mouse and zebrafish, although mutation of zebrafish UHRF1 only results in a milder, late developmental problem, unless maternal stores of the protein are also depleted by injection of morpholino oligonucleotides (46–48). In contrast, *uhrf1*^−^^/^^−^ mouse embryonic stem cells are viable and only exhibit a slight delay in cell cycle progression (45,46). This indicates that, in these cells at least, UHRF1 is not essential for S phase progression. It is not clear whether this is a particular feature of embryonic stem cells or if it is a consequence of cell adaptation to the selective pressure used to generate the homozygous deletion mutant. siRNA knockdown of UHRF1 in NIH-3T3 cells inhibits the replication of heterochromatin in mid-late S phase, leading to the accumulation of cells blocked in early S phase (22). These findings suggest that limiting amounts of UHRF1 are problematic for heterochromatin duplication, although they have little effect on the prior replication of euchromatic regions. It is certainly feasible that UHRF1’s role in replication is confined to heterochromatin duplication in these cells, but it is also possible that very low levels of UHRF1 after siRNA knockdown are capable of supporting the replication of less compacted regions of the genome in early S phase and only become limiting for heterochromatin replication.

It is increasingly being recognized that epigenetic factors can influence replication dynamics by affecting the recruitment of DNA replication proteins. Studies in a variety of eukaryotes have correlated histone acetylation status with replication activity, and most especially with replication licensing, whereas the repressive histone mark H3K9me3 is generally associated with late replicating heterochromatic regions of the genome (49–54). In both humans and *Arabidopsis*, profiling of histone modifications at replication origins has revealed the relative enrichment of H3K4 methylation and H3 acetylation at these regions, suggesting that these chromatin signatures may influence replication initiation (55,56). A further link between epigenetic modification and the replication machinery was recently identified with the finding that histone H4K20me2 is enriched at human replication origins, where it is recognized by the bromo adjacent homology domain of ORC1 (57). Abrogation of H4K20me2 recognition by ORC1 compromises ORC chromatin loading and impairs cell cycle progression in a similar manner to the Uhrf1 depletion we report here. We postulate that H4K20me2 recognition by ORC1 and UHRF1-dependent ORC loading represent co-operative mechanisms to ensure ORC chromatin binding. Interestingly, H4K20me2 recognition by ORC1 appears to be confined to metazoans, where local chromatin structure, rather than DNA sequence alone, is thought to be important in determining replication origins (57–59). In this regard, it is perhaps noteworthy that UHRF1 has been identified in a variety of metazoan species but not in yeast (60).

Two distinct forms of metazoan replication origins have been described (61). At some, replication is reproducibly initiated at a specific defined locus, whereas at others, replication can initiate at one of many potential sites within an initiation zone. It is not clear how individual origins are chosen as initiation sites, although a difference in the underlying chromatin has been recognized as a potential means of specification (61). In somatic cells, a discrete regulatory point within G1 phase, termed the origin decision point, has been identified, at which initiation is directed to occur at specific chromosomal loci rather than randomly distributed sites (62). Interestingly, replication licensing precedes the origin decision point but is not in itself sufficient for origin specification (63). It is clear from our data that a population of Uhrf1 accumulates on chromatin after licensing and is required for some aspect of replication beyond its role in ORC loading. As removal of Uhrf1 after licensing leads to an apparent destabilization of ORC chromatin binding, it is possible that Uhrf1 may have a role in maintaining the pre-IC at licensed origins and could perhaps be important for the process of origin specification at this time. In conclusion, UHRF1 can bind to a number of different epigenetic marks and, through protein interactions, is able to recruit various chromatin modifiers in specific contexts. As such, UHRF1 is able to exert a profound effect on chromatin structure. It therefore represents an attractive candidate for linking chromatin structure with ORC recruitment in the specification of metazoan origins. The identification of the specific histone marks and protein partners involved in this process will be an exciting challenge for the future.

## SUPPLEMENTARY DATA

Supplementary Data are available at NAR Online: Supplementary Figures 1–3 and Supplementary Methods.

## FUNDING

Biotechnology and Biological Sciences Research Council [BBSRC project grant BB\E015662\1]; North West Cancer Research Fund [grant CR 869]. Funding for open access charge: Lancaster University.

*Conflict of interest statement*. None declared.

## Supplementary Material

Supplementary Data
