# Supplementary material for: Depletion of Uhrf1 inhibits chromosomal DNA replication in Xenopus egg extracts
Source: Nucleic Acids Res. 2013 Jun 20;41(16):7725–37. doi: 10.1093/nar/gkt549 (PMC3763540; doi:10.1093/nar/gkt549)
Supplement: Supplementary Data [file supp_41_16_7725__index.html]

Depletion of Uhrf1 inhibits chromosomal DNA replication in Xenopus egg extracts — Depletion of Uhrf1 inhibits chromosomal DNA replication in Xenopus egg extracts — Supplementary Data 

# Depletion of Uhrf1 inhibits chromosomal DNA replication in *Xenopus* egg extracts

## 

files

**Files in this Data Supplement:**

- Supplementary Data - pdf file
