# Supplementary material for: Depletion of Uhrf1 inhibits chromosomal DNA replication in Xenopus egg extracts
Source: Nucleic Acids Res. 2013 Jun 20;41(16):7725–37. doi: 10.1093/nar/gkt549 (PMC3763540; doi:10.1093/nar/gkt549)
Supplement: Supplementary Data [file supp_gkt549_nar-02571-m-2012-File009.pdf]

Figure S1

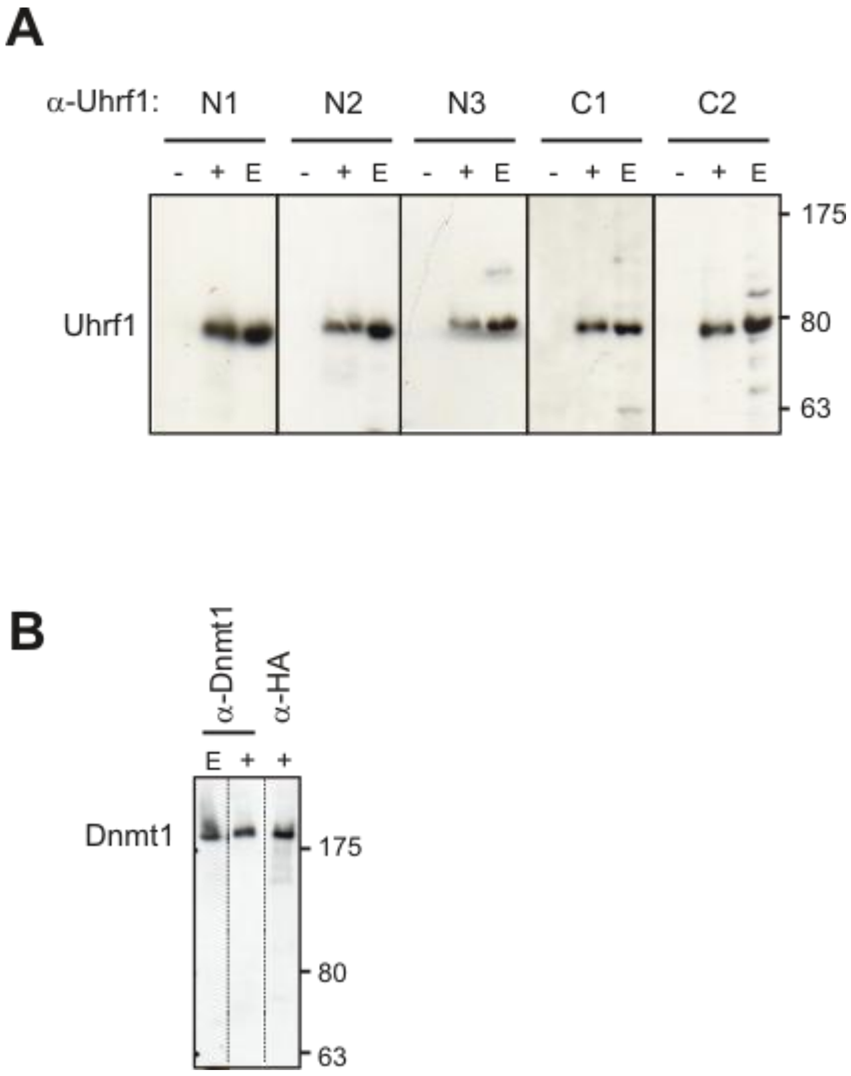

Supplementary Figure 1. Antibody detection of *Xenopus* Uhrf1 and Dnmt1.

(A) Affinity purified antibodies raised to either the N-terminus (N1, N2 and N3) or the C-terminus (C1 and C2) of *Xenopus* Uhrf1 were used for immunodetection of Uhrf1 on Western blots of unprogrammed reticulocyte lysate (-), reticulocyte lysate expressing Uhrf1 (+) and *Xenopus* egg extract (E). (B) Affinity purified antibody raised to *Xenopus* Dnmt1 detects Dnmt1 in *Xenopus* egg extract (E) and HA-tagged Dnmt1 expressed *in vitro* in reticulocyte lysate (+). Anti-HA antibody detection of HA-tagged Dnmt1 serves as a positive control.

Figure S2

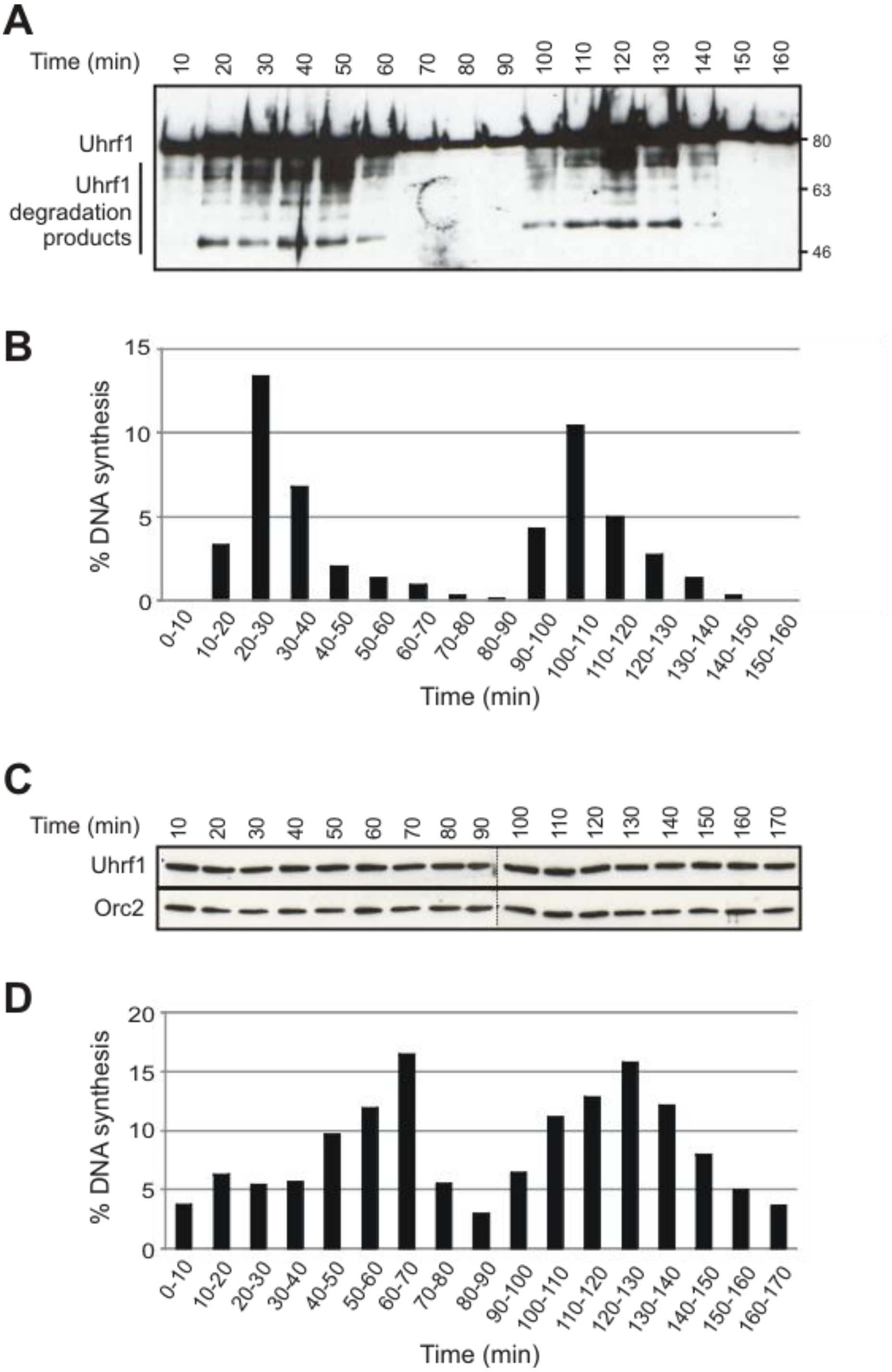

Supplementary Figure 2. S-phase-specific proteolysis of chromatin-associated Uhrf1.

(A) XB extract, without cycloheximide, was supplemented with sperm chromatin and incubated at 21°C. Chromatin was isolated at 10 min intervals and immunoblotted with anti-Uhrf1 antibody. Overexposure reveals Uhrf1 degradation products coincident with S phase (B) Pulse label DNA replication assay performed alongside chromatin timecourse in (A). (C) XB extract, without cycloheximide, was supplemented with sperm chromatin and incubated at 21°C. Uhrf1 levels in total extract were analysed at the indicated times by immunoblotting. (D) Pulse label DNA replication assay for timecourse in (C).

Figure S3

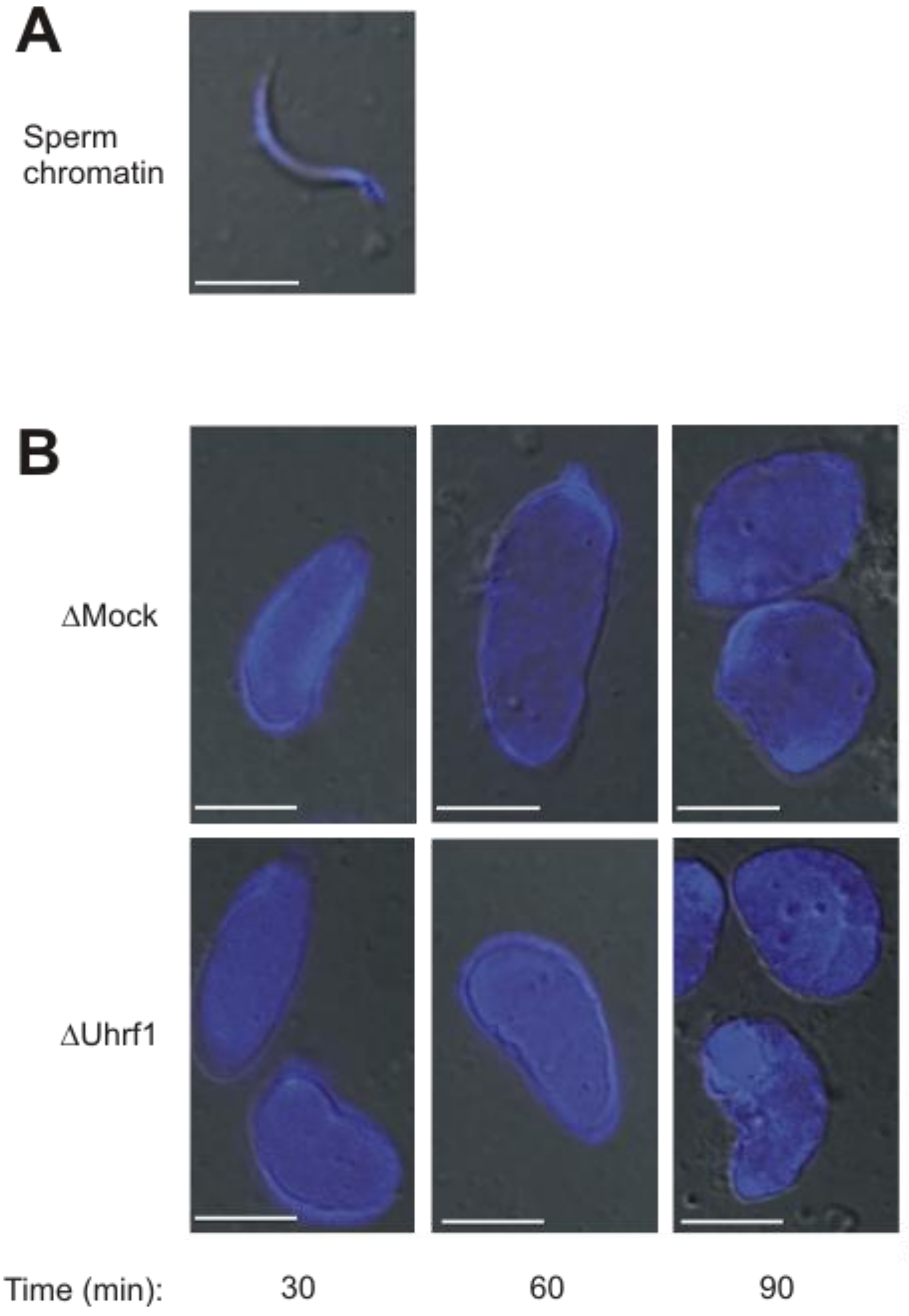

Supplementary Figure 3. Nuclei formation is unaffected by depletion of Uhrf1.

(A) Sperm chromatin, fixed and stained with DAPI (B) XB extract was subjected to two rounds of immunodepletion with mock (ΔMock) or anti-Uhrf1-N2 antibody beads (ΔUhrf1). Following addition of sperm chromatin, extract samples were incubated at 21°C for the indicated times before being fixed and analysed by immunofluorescence microscopy. All scale bars are 10µm.

Supplementary Methods

Antibodies and reagents

In vitro transcription/translation of Uhrf1 and HA-tagged Dnmt1 was performed using a TNT Quick Coupled Transcription/Translation System (Promega) according to the manufacturer's instructions. Anti-HA antibody was purchased from Covance.

Immunofluorsecence microscopy

For analysis of nuclear formation, mock and Uhrf1-depleted egg extracts, supplemented with sperm chromatin, were incubated at 21°C and then fixed in XB buffer containing 3.3% formaldehyde and 0.02μg/ml 4′,6-diamidino-2-phenylindole (DAPI). Images were captured using an Applied Precision DeltaVision Deconvolution microscope system and the software package SoftWoRx. Fluorescence was viewed using the DAPI filter (λEx 360 nm/λEm 457 nm) and reference images were obtained using the differential interference contrast filter. Images were merged using Adobe Photoshop.
